# Effects of hyperuricemia on incident renal replacement therapy and all-cause mortality among patients with chronic kidney disease stages 3-5: a retrospective cohort study

**DOI:** 10.1590/1516-3180.2019.0406211019

**Published:** 2020-03-06

**Authors:** Chia-Lin Lee, Jun-Sing Wang

**Affiliations:** I MD, PhD. Assistant Professor, Department of Medical Research, Taichung Veterans General Hospital, Taichung, Taiwan.; II MD, PhD. Assistant Professor, Department of Internal Medicine, Division of Endocrinology and Metabolism, Taichung Veterans General Hospital, Taichung, Taiwan.

**Keywords:** Mortality, Renal replacement therapy, Uric acid, Chronic kidney disease, End-stage renal disease, Hyperuricemia

## Abstract

**BACKGROUND:**

Findings regarding the effects of hyperuricemia on renal function and mortality have been inconsistent.

**OBJECTIVES:**

To investigate the effects of hyperuricemia on incident renal replacement therapy and all-cause mortality among patients with chronic kidney disease (CKD).

**DESIGN AND SETTING:**

Retrospective cohort study conducted in a medical center in Taiwan.

**METHODS:**

Patients with CKD in stages 3-5, without histories of renal replacement therapy, were consecutively recruited from 2007 to 2013. Their medical history, laboratory and medication data were collected from hospital records. The mean uric acid level in the first year of follow-up was used for analyses. Hyperuricemia was defined as mean uric acid level ≥ 7.0 mg/dl in men or ≥ 6.0 mg/dl in women. The primary outcomes were incident renal replacement therapy and all-cause mortality, and these data were retrospectively collected from hospital records until the end of 2015.

**RESULTS:**

A total of 4,381 patients were analyzed (mean age 71.0 ± 14.8 years; males 62.7%), and the median follow-up period was 2.5 years. Patients with hyperuricemia were at increased risk of incident renal replacement therapy and all-cause mortality, especially those with CKD in stages 4 or 5. Compared with patients with CKD in stage 3 and normouricemia, patients with CKD in stages 4 or 5 presented significantly higher risk of all-cause mortality only if they had hyperuricemia.

**CONCLUSIONS:**

In patients with CKD in stages 3-5, hyperuricemia was associated with higher risk of incident renal replacement therapy and all-cause mortality. Whether treatment with uric acid-lowering drugs in these patients would improve their outcomes merits further investigation.

## INTRODUCTION

Hyperuricemia, defined as a uric acid level higher than 7.0 mg/dl in men or higher than 6.0 mg/dl in women,^1^ has been associated with cardiovascular risk factors^2^^-^^3^ and cardiovascular diseases.^4^^,^^5^ Hyperinsulinemia, a known consequence of insulin resistance, has been shown to reduce urinary excretion of uric acid.^6^ Meanwhile, uric acid retention due to renal vasoconstriction is common in patients with hypertension.^7^ In a meta-analysis on 25 observational studies with nearly 100,000 participants,^8^ it was demonstrated that hyperuricemia independently increased the risk of incident hypertension. In another meta-analysis on 17 prospective observational studies with more than 160,000 participants,^9^ a uric acid level that was one standard deviation higher was associated with an odds ratio for coronary heart disease of 1.07 (95% confidence interval, CI 1.04-1.10).

With regard to the risk of kidney disease, hyperuricemia has been associated with incident chronic kidney disease (CKD). In a prospective cohort study on more than 21,000 healthy volunteers,^10^ a uric acid level ≥ 7.0 mg/dl at baseline was associated with a higher risk of incident kidney disease (defined as an estimated glomerular filtration rate [eGFR] < 60 ml/min/1.73 m^2^) during a median follow-up duration of 7 years. Similar findings were noted in another prospective study^11^ on more than 13,000 participants pooled from two community-based cohorts with a mean follow-up duration of 8.5 years. These results were consistent with the findings from a meta-analysis^12^ on 15 cohort studies with a total of nearly 100,000 relatively healthy individuals and nearly 3,500 incident CKD cases (defined as eGFR < 60 ml/min/1.73 m^2^).

However, among patients with CKD who were not on dialysis, the data regarding the effects of hyperuricemia on renal function and mortality have been limited and the findings have been inconsistent. Among patients with CKD in stages 3-4 who were on chronic allopurinol therapy for hyperuricemia,^13^ withdrawal of allopurinol led to a significant increase in serum uric acid level and a significant deterioration in renal function, especially in those who were not on blockers of the renin-angiotensin system. In contrast, uric acid was not associated with the rate of decline in renal function in another cohort study^14^ on patients with CKD in stages 3-5. With regard to risk of mortality, hyperuricemia was an independent risk factor for all-cause and cardiovascular mortality (but not kidney failure) in a cohort of patients with CKD in stages 3-4 during a median follow-up of 10 years.^15^ In contrast, hyperuricemia was not associated with cardiovascular diseases and all-cause mortality in another cohort of patients with CKD in stages 3-4 during a median follow-up of 9 years.^16^

## OBJECTIVE

In this study, we aimed to investigate the effects of hyperuricemia on incident renal replacement therapy (RRT) and all-cause mortality, among patients with CKD in stages 3-5 who were not on dialysis at the baseline.

## METHODS

This was a retrospective cohort study conducted in a medical center in central Taiwan. Patients with CKD in stages 3-5 with no history of RRT were consecutively recruited in a CKD care program from January 2007 to December 2013. The patients’ medical history, laboratory and medication data were collected from hospital records. The primary outcomes of this study were incident RRT and all-cause mortality, and these data were collected from hospital records until the end of 2015. We included patients for whom information about their vital status (records of follow-up visits or mortality events) were available in the analyses. This study was approved by the Local Institutional Review Board in Taichung, Taiwan (approval number CE16253A; approval date Oct 21, 2016). This report complied with the STROBE (Strengthening the Reporting of Observational Studies in Epidemiology) statement.

Baseline eGFR was determined using the Modification of Diet in Renal Disease (MDRD) equation (GFR = 186.3 × serum creatinine^−1.154^ × age^−0.203^ × 0.742 if female × 1.21 if black).^17^ We divided our patients into CKD in stages 3-5 according to their baseline eGFR (CKD stage 3 = eGFR 30 to < 60 ml/min/1.73 m^2^; CKD stage 4 = eGFR 15 to < 30 ml/min/1.73 m^2^; and CKD stage 5 = eGFR < 15 ml/min/1.73 m^2^ with no RRT). The patients’ systolic and diastolic blood pressure and uric acid levels were collected from hospital records, and the mean blood pressure and uric acid levels in the first year of follow-up were used for analyses. Hyperuricemia was defined as a mean uric acid level ≥ 7.0 mg/dl in men or ≥ 6.0 mg/dl in women. The uric acid-lowering drugs used included allopurinol and benzbromarone.

RRT was defined as hemodialysis, peritoneal dialysis or renal transplantation. We classified patients’ cause of death into cardiovascular or non-cardiovascular death^18^ by reviewing the medical records. Cardiovascular death included sudden cardiac death, death due to acute myocardial infarction, death due to heart failure, death due to stroke and death due to other cardiovascular causes. The codes for cardiovascular deaths that we used were International Classification of Diseases (ICD)-9 390-448. Non-cardiovascular death was defined as any death not covered by cardiac death or vascular death.^18^ All causes of death were independently reviewed by two board-certified physicians of internal medicine. In the event of an inconsistency, an independent cardiologist reviewed the medical records and made the final classification.

All of the statistical analyses were performed using the SAS software (version 9.4; SAS Institute, Inc., Cary, NC, USA). Continuous variables were reported as the mean ± standard deviation (SD), while categorical data were given as numbers (with percentages). The differences in clinical variables between groups were tested for statistical significance using the chi-square test for categorical variables; or using an independent-sample t test or one-way analysis of variance (ANOVA) for continuous variables.

We used Cox’s proportional hazards model to compare the hazard ratios (HRs) of RRT, all-cause mortality and cardiovascular and non-cardiovascular death among patients with different CKD stages or uric acid levels. Since mortality was an event that competed with RRT, an extended Cox’s proportional hazards model was used to calculate the subdistribution hazard ratio (SHR) as a sensitivity test.^19^ For cause-specific mortality, we considered non-cardiovascular death to be an event competing with cardiovascular death, and vice versa. Therefore, an extended Cox’s proportional hazards model was used to determine the SHR of cardiovascular and non-cardiovascular death among patients with different CKD stages or uric acid levels. The variables that were adjusted for in the analyses included factors that were known to be associated with the risk of mortality or with uric acid levels (age, sex, systolic blood pressure, smoking history, diabetes and use of uric acid-lowering drugs and statins). In all of the statistical analyses, a two-sided P-value < 0.05 was considered statistically significant.

## RESULTS

A total of 4,381 patients were included in the analyses (mean age 71.0 ± 14.8 years; males 62.7%), and the median follow-up period was 2.5 years. Table 1 shows the baseline characteristics of the study patients according to CKD stages and uric acid levels. The patients who were at a later stage of CKD were younger, were more frequently female, had higher systolic and diastolic blood pressure, were less likely to have a smoking history, were more likely to have diabetes, had higher uric acid levels and were less likely to be on a statin, compared with those at CKD stage 3. The patients who had hyperuricemia (defined as a mean uric acid level ≥ 7.0 mg/dl in men or ≥ 6.0 mg/dl in women) were younger, were more frequently female, had higher systolic and diastolic blood pressure, were less likely to have a smoking history, had lower eGFR and were more likely to be on uric acid-lowering treatment, compared with those presenting normouricemia.

**Table 1.** Baseline characteristics of study patients according to CKD stages and uric acid levels

Variables	Overall	CKD stages				Uric acid levels		
		Stage 3	Stage 4	Stage 5	P	Normouricemia	Hyperuricemia^a^	P
Number of patients	4381	2076	1365	940		1151	3230	
Age, years	71.0 ± 14.8	72.2 ± 14.4	71.5 ± 15.0	67.7 ± 14.9	< 0.001	73.4 ± 13.8	70.2 ± 15.1	< 0.001
Male, n (%)	2747 (62.7)	1491 (71.8)	794 (58.2)	462 (49.2)	< 0.001	864 (75.1)	1883 (58.3)	< 0.001
Systolic BP, mm Hg^b^	134 ± 16	132 ± 15	134 ± 16	137 ± 17	< 0.001	133 ± 19	135 ± 19	0.041
Diastolic BP, mm Hg^b^	74 ± 9	74 ± 9	74 ± 10	76 ± 9	< 0.001	74 ± 11	75 ± 11	0.039
Smoking history, n (%)	1688 (38.5)	880 (42.4)	506 (37.1)	302 (32.1)	< 0.001	501 (43.5)	1187 (36.8)	< 0.001
Diabetes, n (%)	1776 (40.5)	784 (37.8)	614 (45.0)	378 (40.2)	< 0.001	479 (41.6)	1297 (40.2)	0.386
eGFR, ml/min/1.73 m^2^	28.1 ± 13.2	39.8 ± 6.4	23.1 ± 4.9	9.6 ± 3.4	< 0.001	31.8 ± 12.4	26.7 ± 13.3	< 0.001
Uric acid, mg/dl^b^	8.0 ± 2.1	7.6 ± 1.8	8.2 ± 2.3	8.5 ± 2.2	< 0.001	5.7 ± 0.9	8.7 ± 1.8	< 0.001
Uric acid-lowering drugs, n (%)	1229 (28.1)	556 (26.8)	403 (29.5)	270 (28.7)	0.189	279 (24.2)	950 (29.4)	< 0.001
Statins, n (%)	1523 (34.8)	768 (37.0)	492 (36.0)	263 (28.0)	< 0.001	383 (33.3)	1140 (35.3)	0.217

Continuous variables are expressed as mean ± standard deviation. Categorical data are presented as numbers (with percentages). ^a^Defined as a mean uric acid level ≥ 7.0 mg/dl in men or ≥ 6.0 mg/dl in women. ^b^Mean level within the first year of follow-up.

BP = blood pressure; CKD = chronic kidney disease; eGFR = estimated glomerular filtration rate.

Overall, 1229 patients (28.1%) were undergoing treatment with uric acid-lowering drugs. They were more frequently male (75.2% versus 57.8%; P < 0.001), more likely to have a smoking history (45.5% versus 35.8%; P < 0.001), less likely to have diabetes (33.6% versus 43.2%; P < 0.001) and had higher uric acid levels (8.4 ± 2.1 versus 7.8 ± 2.1 mg/dl; P < 0.001), compared with those who were not on uric acid-lowering treatment.

Table 2 shows the effects of CKD and uric acid levels on incident RRT and mortality. During a median follow-up period of 2.5 years, 21.3% of the patients (n = 932) received RRT, while the mortality rate was 8.1% (n = 356). Compared with patients at CKD stage 3, those at CKD stage 4 or stage 5 presented significantly higher risk of incident RRT (all P < 0.001). The findings remained significant when multiple factors were adjusted for (all P < 0.001) and the competing risk of mortality was considered (all P < 0.001). Similar findings were noted when patients with hyperuricemia were compared with those with normouricemia, with regard to the risk of incident RRT. Patients at a later stage of CKD were also at higher risk of all-cause mortality, as well as cardiovascular and non-cardiovascular death, compared with those at CKD stage 3. Similarly, patients with hyperuricemia were at higher risk of all-cause mortality and non-cardiovascular death, compared with normouricemic patients (Table 2).

**Table 2.** Cox proportional hazards models for renal replacement therapy and mortality according to CKD stages and uric acid levels

	Renal replacement therapy (n = 932)							
	Univariate HR (95% CI)			P	Multivariate^a^ HR (95% CI)	P	Multivariate^b^ SHR (95% CI)	P
CKD stages								
Stage 3	1.00 (reference)				1.00 (reference)		1.00 (reference)	
Stage 4	4.13 (3.32-5.14)			< 0.001	4.34 (3.29-5.71)	< 0.001	4.10 (3.14-5.35)	< 0.001
Stage 5	22.43 (18.29-27.52)			< 0.001	24.23 (18.66-31.47)	< 0.001	21.68 (16.74-28.08)	< 0.001
Uric acid levels								
Normouricemia	1.00 (reference)				1.00 (reference)		1.00 (reference)	
Hyperuricemia	2.12 (1.78-2.53)			< 0.001	1.71 (1.37-2.13)	< 0.001	1.60 (1.26-2.04)	< 0.001
	All-cause mortality (n = 356)				CV death (n = 65)		Non-CV death (n = 291)	
	Univariate HR (95% CI)	P	Multivariate^a^ HR (95% CI)	P	Multivariate^a^ SHR (95% CI)	P	Multivariate^a^ SHR (95% CI)	P
CKD stages								
Stage 3	1.00 (reference)		1.00 (reference)		1.00 (reference)		1.00 (reference)	
Stage 4	1.61 (1.27-2.04)	< 0.001	1.88 (1.42-2.48)	< 0.001	2.58 (1.28-5.22)	0.008	1.72 (1.27-2.32)	< 0.001
Stage 5	1.56 (1.19-2.05)	0.002	1.81 (1.28-2.58)	< 0.001	2.82 (1.19-6.68)	0.018	1.61 (1.09-2.39)	0.016
Uric acid levels								
Normouricemia	1.00 (reference)		1.00 (reference)		1.00 (reference)		1.00 (reference)	
Hyperuricemia	1.21 (0.95-1.55)	< 0.001	1.58 (1.16-2.15)	0.004	1.61 (0.72-3.59)	0.246	1.56 (1.12-2.17)	0.009

^a^Adjusted for age, gender, systolic blood pressure, smoking history, diabetes and use of uric acid-lowering drugs and statins. ^b^Considering the competing risk of mortality in addition to multivariate adjustment. ^c^Defined as a mean uric acid level ≥ 7.0 mg/dl in men or ≥ 6.0 mg/dl in women. CKD = chronic kidney disease; CV = cardiovascular; HR = hazard ratio; SHR = subdistribution hazard ratio.

The effects of hyperuricemia on incident RRT and mortality across the CKD stages are shown in Table 3. The higher risk of incident RRT associated with hyperuricemia was statistically significant among patients at CKD stage 4 or stage 5, but not among patients at CKD stage 3. With regard to risk of mortality, hyperuricemia was associated with significantly higher risk of all-cause mortality among patients at CKD stage 4. Patients with hyperuricemia did not have significantly higher risk of cardiovascular death across the CKD stages. Hyperuricemia was associated with significantly higher risk of non-cardiovascular death among patients at CKD stage 4 or stage 5 (Table 3).

**Table 3.** Cox proportional hazards model for renal replacement therapy and mortality according to uric acid levels at different CKD stages

		Renal replacement therapy						
		Univariate HR (95% CI)	Multivariate^a^ HR (95% CI)	P	Multivariate^b^ SHR (95% CI)		P	
CKD stage 3	Normouricemia	1.00 (reference)	1.00 (reference)		1.00 (reference)			
	Hyperuricemia^c^	1.52 (0.99-2.32)	1.31 (0.76-2.26)	0.331	1.28 (0.75-2.22)		0.368	
CKD stage 4	Normouricemia	1.00 (reference)	1.00 (reference)		1.00 (reference)			
	Hyperuricemia^c^	1.89 (1.35-2.65)	1.77 (1.15-2.72)	0.010	1.68 (1.10-2.57)		0.016	
CKD stage 5	Normouricemia	1.00 (reference)	1.00 (reference)		1.00 (reference)			
	Hyperuricemia^c^	1.61 (1.26-2.04)	1.62 (1.20-2.17)	0.002	1.52 (1.12-2.06)		0.007	
		All-cause mortality			CV death		Non-CV death	
		Univariate HR (95% CI)	Multivariate^a^ HR (95% CI)	P	Multivariate^a^ SHR (95% CI)	P	Multivariate^a^ SHR (95% CI)	P
CKD stage 3	Normouricemia	1.00 (reference)	1.00 (reference)		1.00 (reference)		1.00 (reference)	
	Hyperuricemia^c^	1.08 (0.75-1.55)	1.31 (0.86-2.01)	0.214	2.87 (0.62-13.25)	0.176	1.18 (0.75-1.85)	0.470
CKD stage 4	Normouricemia	1.00 (reference)	1.00 (reference)		1.00 (reference)		1.00 (reference)	
	Hyperuricemia^c^	1.00 (0.67-1.50)	1.70 (1.02-2.85)	0.043	1.28 (0.41-3.99)	0.667	1.80 (1.01-3.22)	0.047
CKD stage 5	Normouricemia	1.00 (reference)	1.00 (reference)		1.00 (reference)		1.00 (reference)	
	Hyperuricemia^c^	1.76 (0.88-3.52)	2.48 (0.89-6.95)	0.083	0.76 (0.14-4.25)	0.756	4.23 (1.02-17.50)	0.047

^a^Adjusted for age, gender, systolic blood pressure, smoking history, diabetes and use of uric acid-lowering drugs and statins. ^b^Considering the competing risk of mortality in addition to multivariate adjustment. ^c^Defined as a mean uric acid level ≥ 7.0 mg/dl in men or ≥ 6.0 mg/dl in women.

CKD = chronic kidney disease; HR = hazard ratio; CV = cardiovascular; SHR = subdistribution hazard ratio.

When we stratified our patients according to their CKD stages and uric acid levels (Table 4), the weighted event rate of incident RRT per 1000 person-years increased from 13.75 for patients at CKD stage 3 with normouricemia to 398.43 for patients at CKD stage 5 with hyperuricemia. With regard to the risk of mortality among patients with normouricemia, those at CKD stage 4 or stage 5 did not have significantly higher risk of all-cause mortality, compared with patients at CKD stage 3. In contrast, patients at CKD stage 4 or stage 5 with hyperuricemia had significantly higher risk of all-cause mortality, as well as cardiovascular and non-cardiovascular death, compared with those at CKD stage 3 with normouricemia. These findings remained consistent after adjustment for multiple factors (Table 4).

**Table 4.** Cox proportional hazards model for all-cause mortality and for CV and non-CV deaths

Uric acid levels	CKD stages	All-cause mortality				CV death		Non-CV death	
		Number of events	Weighted event rate^a^	Univariate HR (95% CI)	Multivariate^b^ HR (95% CI)	Multivariate^b^ SHR (95% CI)	P	Multivariate^b^ SHR (95% CI)	P
Normouricemia	Stage 3	42	20.34	1.00 (reference)	1.00 (reference)	1.00 (reference)		1.00 (reference)	
	Stage 4	31	34.30	1.69 (1.06-2.68)	1.62 (0.90-2.90)	5.05 (0.92-27.83)	0.063	1.34 (0.71-2.53)	0.375
	Stage 5	9	20.54	1.01 (0.49-2.07)	1.01 (0.35-2.88)	7.86 (1.00-61.74)	0.050	0.52 (0.12-2.27)	0.387
Hyperuricemia^c^	Stage 3	94	21.91	1.08 (0.75-1.55)	1.36 (0.89-2.08)	3.10 (0.68-14.17)	0.144	1.23 (0.79-1.93)	0.363
	Stage 4	107	34.52	1.70 (1.19-2.43)	2.66 (1.75-4.05)	6.68 (1.52-29.38)	0.012	2.28 (1.46-3.58)	< 0.001
	Stage 5	73	36.14	1.78 (1.22-2.60)	2.73 (1.70-4.36)	7.15 (1.52-33.66)	0.013	2.33 (1.40-3.87)	0.001

^a^Per 1000 person-years. ^b^Adjusted for age, gender, systolic blood pressure, smoking history, diabetes and use of uric acid-lowering drugs and statins. ^c^Defined as a mean uric acid level ≥ 7.0 mg/dl in men or ≥ 6.0 mg/dl in women.

CKD = chronic kidney disease; CV = cardiovascular; HR = hazard ratio; SHR = subdistribution hazard ratio.

## DISCUSSION

In this study, we investigated patients with CKD in stages 3-5 and found that patients with hyperuricemia (defined as a mean uric acid level ≥ 7.0 mg/dl in men or ≥ 6.0 mg/dl in women) were at increased risk of incident RRT and all-cause mortality, independent of traditional risk factors such as age, gender, blood pressure, smoking, diabetes etc. (Table 2). Hyperuricemia was associated with worse outcomes mainly among patients at CKD stage 4 or stage 5 (significantly higher risk of incident RRT and non-cardiovascular mortality; Table 3).

Patients with advanced CKD and hyperuricemia were at substantially increased risk of incident RRT. Moreover, compared with patients with CKD in stage 3 with normouricemia, patients at CKD stage 4 or stage 5 were at significantly higher risk of all-cause mortality only if they had hyperuricemia (Table 4).

Our findings are consistent with those of previous studies^10^^,^^11^^,^^12^^,^^20^^,^^21^ that showed that hyperuricemia was an independent risk factor for decreased kidney function and presence of end-stage renal disease in general populations. Our results suggest that hyperuricemia was a risk factor for decreased kidney function requiring RRT among patients with CKD in stages 3-5. Several studies have reported that hyperuricemia was not associated with any decline in renal function^14^ or kidney failure^14^^,^^15^ among patients with CKD in stages 3-5.

Certain factors may account for these inconsistent findings. First, because uric acid is excreted by the kidney, a decrease in renal function is inevitably accompanied by an increase in serum uric acid level. This complicating phenomenon therefore makes it particularly challenging to study the role of uric acid in CKD. Second, patients with advanced CKD and/or hyperuricemia are at increased risk of mortality.^22^^,^^23^^,^^24^^,^^25^^,^^26^ Thus, the lack of association between hyperuricemia and decreased kidney function in previous studies might be explained by the fact that the competing risk of mortality was not considered. In our study, we demonstrated that hyperuricemia was associated with incident RRT among patients with CKD (Table 2), especially those at CKD stage 4 or stage 5 (Table 3). Moreover, the risk remained significant after adjustment for multiple factors and consideration of the competing risk of mortality (Tables 2 and 3).

We also reported that hyperuricemia was associated with a higher risk of all-cause mortality (mainly non-cardiovascular death) among patients with CKD in stages 3-5 (Table 2). It is well known that patients with CKD are at increased risk of mortality and cardiovascular events.^22^^,^^23^^,^^24^ Although occurrences of hyperuricemia had previously been correlated with risks of mortality and cardiovascular diseases,^25^^,^^26^ conflicting data regarding this association had been reported in the general population^26^^,^^27^^,^^28^ and among patients with diabetes^29^^,^^30^ or CKD.^15^^,^^16^ Our data support an association between hyperuricemia and higher risk of mortality among patients with CKD.^15^^,^^31^^,^^32^

Interestingly, less than 20% of the all-cause mortality comprised cardiovascular death among our patients (Table 2). Although patients with CKD are at increased risk of cardiovascular events,^22^^,^^23^^,^^24^ it is worth noting that the majority of patients with CKD have died due to non-cardiovascular causes.^33^^,^^34^ For example, in a large population of non-dialysis-dependent CKD patients,^33^ less than 35% of the all-cause mortality was due to cardiovascular death during a median follow-up duration of 2.3 years. In another Asian population with CKD,^34^ less than 20% of the all-cause mortality was due to cardiovascular death among patients with baseline eGFR < 45 ml/min/1.73 m^2^, over the course of a median follow-up duration of 9.8 years. Thus, most patients with CKD died due to non-cardiovascular causes.^34^ We found that hyperuricemia was associated with higher risk of all-cause mortality and non-cardiovascular death among patients with CKD in stages 3-5 (Table 2).

In line with previous reports,^33^^,^^34^ the leading cause of non-cardiovascular death in our patients was malignancy (n = 202; 56.7% of the all-cause mortality). We found that hyperuricemia was associated with higher risk of non-cardiovascular death (Table 2), especially among patients with CKD in stage 4 or stage 5 (Table 3).

More than three decades ago, it was hypothesized that uric acid might protect against carcinogenesis, owing to its antioxidant properties.^35^ However, studies in more recent decades have suggested that hyperuricemia was associated with higher risk of incidence of cancer and mortality.^36^ Thus, despite the possible antioxidative effects of uric acid, this substance may play a contributory role in carcinogenesis. Since reactive oxygen species play a critical role in cell growth and survival, in both normal and cancer cells,^37^ uric acid may promote cancer cell growth and survival by scavenging reactive oxygen species and reducing oxidative stress-induced apoptosis.^36^^,^^37^

Another mechanism through which hyperuricemia may be related to higher risk of incidence of cancer and mortality is inflammation. Uric acid has been found to induce the expression of several inflammatory mediators (such as monocyte chemoattractant protein-1 and C-reactive protein),^38^ which may lead to a microenvironment with low-grade inflammation,^36^ thus favoring transformation into cancer cells.

Taken together, a growing body of evidence suggests that hyperuricemia is associated with a higher risk of mortality due to cancer,^36^ which is the leading cause of non-cardiovascular death among patients with CKD.^33^^,^^34^ Our results suggest that hyperuricemia was associated with higher risk of non-cardiovascular death among patients with non-dialysis-dependent CKD.

Our study had some limitations. First, the causal relationship between hyperuricemia and incident RRT and mortality among patients with CKD could not be confirmed in this cohort study. Although several small studies have reported that treatment with the uric acid-lowering drug allopurinol slowed the progression of kidney disease and reduced cardiovascular risk in patients with CKD,^39^^,^^40^ this effect needs to be confirmed in a large-scale prospective study. Second, the events of RRT and mortality among our patients were collected from our hospital records.

Third, this was a retrospective cohort study. Thus, we may have underestimated the event rates among our patients. In a large prospective cohort study^24^ in which the mortality data were obtained from death certificate codes in Taiwan, the all-cause mortality rate among patients with CKD in stages 3-5 (n = 26,757) was 16.2% over a median follow-up period of 7.5 years. The all-cause mortality rate among our patients was 8.1% over a median follow-up period of 2.5 years. Although patients with CKD in stages 3-5 in the aforementioned study^24^ were younger (mean age around 61.9 years) than our patients (mean age 71.0 years), the extent of underestimation of mortality events in our study was likely to have been small, and therefore probably did not confound the results.

Lastly, we investigated patients with CKD in stages 3-5 in this study. Whether our findings may be generalized to patients with early stages of CKD needs further investigation.

## CONCLUSION

We demonstrated that in patients with CKD in stages 3-5, hyperuricemia was associated with higher risk of incident RRT and all-cause mortality (mainly non-cardiovascular death). Further studies are needed, to investigate whether treatment with uric acid-lowering drugs in such patients would improve their outcomes.
